# Filtration Scheduling: Quality Changes in Freshly Produced Virgin Olive Oil

**DOI:** 10.3390/foods9081067

**Published:** 2020-08-06

**Authors:** Lorenzo Guerrini, Carlotta Breschi, Bruno Zanoni, Luca Calamai, Giulia Angeloni, Piernicola Masella, Alessandro Parenti

**Affiliations:** Department of Agriculture Food, Environment and Forestry (DAGRI), Università degli Studi di Firenze, 50121 Florence, Italy; lorenzo.guerrini@unifi.it (L.G.); bruno.zanoni@unifi.it (B.Z.); luca.calamai@unifi.it (L.C.); giulia.angeloni@unifi.it (G.A.); piernicola.masella@unifi.it (P.M.); alessandro.parenti@unifi.it (A.P.)

**Keywords:** aroma kinetics, filtration timing, flavour, fusty, veiled olive oil

## Abstract

Filtration is the most widespread stabilisation operation for extra virgin olive oil, preventing microbial and enzymatic changes. However, during the harvest, the workload of olive mills is at its peak. This results in two approaches to filtration: (i) delays it until after harvesting, increasing the risk of degraded oil quality, and (ii) filters it immediately, increasing the workload. The aim of our experiment is to assess the risk of delaying filtration and establish a safe delay time. Changes in the sensory profile and volatile compound contents were evaluated during 30 days in filtered and unfiltered samples. Significant differences were related to filtration: both turbidity grade and microbial contamination; no differences for the legal parameters were found. Two, contrasting, results were obtained with respect to oil quality: (i) the fusty defect, appearing in less than five days in unfiltered oils, leading to the downgrade of the oil’s commercial category, and (ii) filtration removing some lipoxygenase volatile compounds. Consequently, a fruity attribute was more pronounced in unfiltered samples until day five of storage; it seems that, from this point, the fusty defect masked a fruity attribute. Hence, filtering within a few days strongly reduced the risk of degraded oil quality compared to a delayed filtration.

## 1. Introduction

Olive oil is composed of triacylglycerols, which make up over 98% of the total weight and about 2% minor components—aliphatic and triterpene alcohols, hydrocarbons, sterols, non-glyceride esters, pigments, lipophilic and hydrophilic phenols, and volatile compounds [1].

Volatile compounds are a complex mix of aldehydes, alcohols, ketones, acids, hydrocarbons, and esters and are closely associated with both oil flavour and its positive and negative sensory attributes [2,3]. Flavour is not only a key characteristic that affects consumer preferences [2,4], but it is also a quality parameter in oil classification. In 1991, the European Commission laid down legal limits for many quality parameters, including sensory attributes [5]. The panel has to confirm the absence of sensory defects (median of defect equal to 0) and the presence of the positive, fruity attribute (fruity median greater than 0) in order to classify an olive oil as “extra virgin”. On the other hand, when the median of the defects is between 0 and 3.5, and the fruity median is greater than 0, the olive oil is classified as “virgin”; when the median of the defects is greater than 3.5, the olive oil is classified as “lampante” [6]. Five sensory defects, in particular, are described in the official sensory evaluation methodology: fusty, muddy, mustiness-humidity, winey-vinegary, and rancid.

It is well-known that the volatile compound content of olive oil is affected by several operating factors. The first group relate to “in-field” factors such as the environment, agronomy, genetics, timing, and type of harvesting; then, there are “out-of-field” factors, such as the transport and storage of fruit; operating conditions during extraction; and oil storage, packaging, and transport conditions [7,8,9,10,11].

The pleasant, fruity attribute of extra virgin olive oil (EVOO) is mainly related to lipoxygenase (LOX) pathway volatile compounds [12,13]. Z-3-hexenal and E-2-hexenal compounds are described as having “green leaves” and “green and sweet” sensory notes, respectively. Their low odour thresholds mean that they are the most important volatile compounds in the LOX pathway [8,12,13,14], along with several others that contribute to the fruity attribute.

The rancid attribute is a widely studied sensory defect. It is due to lipid auto-oxidation molecules, generally heptane, E-2-heptenal, 2,4-heptadienal, 2-heptanol, nonanal, 2,4-nonadienal, and decanal volatile compounds [2,8]. During oil storage, oxidation is promoted by factors such as light (photo-oxidation), temperature, and minor components such as metals and pigments. Furthermore, it is well-studied that olive oil antioxidant phenolic compounds (i.e., especially the secoiridoids) are able to slow down rancid defect formations [7,15].

The remaining defects (fusty, muddy, mustiness-humidity, and winey-vinegary) are commonly related to microbial spoilage [16]. Incorrect fruit storage conditions (long period of time, high relative humidity, and high temperature) and poor hygiene during oil extraction support microbial activity and growth. Various sensory defects have been related to different microorganism genera, with a predominance of yeasts and moulds [7,16,17]. Volatile compounds such as 2-methyl-butanal, 3-methyl-butanal, isobutanol, 6-methyl-5-hepten-2-one, 2-octanol, 2-heptanone, 2-nonanone, and many others have been described as having sensory notes of “muddy sediment”, “earthy”, “mushroom”, “oily”, “winey”, and “vinegar” [2,17,18,19].

Extra virgin olive oil producers aim to ensure that the quality characteristics of the extracted oil remain as stable as possible over time. Typical operations to achieve this objective include finishing centrifugation, oil clarification, and filtration; they transform the appearance of the oil from veiled to limpid, either individually or in combination. Although it is well-known that these operations stabilise the olive oil quality, many producers and consumers prefer unfiltered olive oil, because it is considered “less processed”, “greener”, “healthier”, and “whole” [16]. For these reasons, several unfiltered olive oils can be found in commerce. The veiled appearance of unfiltered olive oil is due to the presence of suspended material. The latter is a combination of microdroplets of vegetation water and insoluble fruit solids, which are responsible for a wide spectrum of “turbidities” [20,21,22]. Veiled olive oils are rich in microflora, notably yeasts of candida species [11,16,22,23,24,25]. The presence of these solid particles, which are rich in sugars, proteins, and water droplets, is associated with high water activity (greater than 0.6) [24]. This, in turn, supports microorganism and enzyme activity and is responsible for the development of sensory defects such as the fusty attribute [25].

Filtration, when applied, is typically used to obtain a limpid olive oil, as it is an efficient way to completely remove water in emulsion and solids in suspension [11,13,26,27,28]. There are currently two filtration scheduling approaches. Some extra virgin olive oil producers are used to filter olive oil in-line with the milling process, while others wait until the end of the harvesting season. The former approach aims to minimise the risk of olive oil spoilage but increases the mill’s workload. Conversely, the latter approach does not increase the workload but does expose the olive oil to the risk of spoilage.

To the best of our knowledge, there is a lack of data regarding the degradation rate of olive oil and, consequently, the optimal moment for filtration. Thus, the aim of this study is to assess the effects of the delayed depth filtration of veiled olive oil on volatile compound contents and the presence of sensory defects that downgrade oil from the “extra virgin” to “virgin” quality category. For this investigation, during the first month of storage in protective conditions, we have examined the kinetics of volatile compound contents, and sensory analyses, in immediately filtered olive oil samples and in the respective veiled olive oil samples, which would be hypothetically filtered after several days, as often happens during olive oil production.

## 2. Materials and Methods

### 2.1. Olive Oil Samples

Olive oil samples were processed during the month of November 2018 at Frantoio L’Antellino (Bagno a Ripoli, Florence, Italy). The olive oil plant was provided with an olive cleaner machine (Pegaso 500, Officine Meccaniche Toscane, Bagno a Ripoli, Florence, Italy), followed by a blade cutter crusher (model FR.350, Mori-TEM Srl, Tavarnelle, Florence, Italy). The olive paste was kneaded in a sealed vertical malaxer (Officine Meccaniche Toscane, Bagno a Ripoli, Florence, Italy), of 500 kg capacity at room temperature. Then, two three-phase decanters with a nominal working capacity of 1500 kg/h (X15 D.E., Officine Meccaniche Toscane, Bagno a Ripoli, Florence, Italy) were used for the olive oil extraction.

Veiled oil samples were collected immediately after extraction, with no mechanical separation, while samples were processed using a filter press (Mori-TEM Srl, Tavarnelle, Florence, Italy). The filter press was equipped with eleven clarifying disposable filter sheets (CKP V8, Cordenons S.p.A., Milano, Italy). A 40 cm × 40-cm plate filter press was used. The device, equipped with eleven V8 clarifying disposable filter sheets (Gruppo Cordenons S.p.A., Pordenone, Italy), was used. The technical specifications of the plate filter press were: nominal cut-off filtration, 12 µm; thickness, 3.75 mm; nominal flow rate, 160 L min^−1^ m^−2^; and weight, 1050 g m^−2^. Oil samples were immediately characterised in terms of turbidity grade, water and insoluble solids content, water activity, peroxide values, acidity, UV spectroscopic indexes, microbial cell count, and volatile compound content. Peroxide value, acidity, UV spectroscopic indexes, and microbial cell counts were also measured after 30 days of storage. Volatile compounds and sensory attributes were measured by a panel test after 0.25, 1, 2, 3, 4, 5, 10, 15, and 30 days of storage. Three olive fruit batches (cv. Frantoio) were used as replicates.

Two-hundred and fifty millilitre green bottles were used to store the olive oil samples at room temperature and dark conditions. The olive oil samples were bottled by hand. At the designated times, bottles were shaken for 1 min, opened, and olive oil samples were analysed by the panel test. At the same time, 12 mL of each oil sample was placed into a 15 mL sealed vial, frozen, and then stored at −18 °C before further analyses.

### 2.2. Chemicals and Reagents

All chemicals were of analytical reagent grade. Chloroform, phenolphthalein, and orthophosphoric acid were supplied by Merck KGaA (Darmstadt, Germany). Acetic acid glacial, potassium iodide, and sodium thiosulfate were supplied by Nova Chimica Srl (Milan, Italy). Starch, isooctane, sodium hydroxide, and ethanol were supplied by CARLO ERBA Reagents Srl (Milan, Italy). All chemicals and standards used for volatile compound measurements were of analytical reagent grade and purchased from Sigma-Aldrich (Steinheim, Germany).

### 2.3. Chemical Analyses

According to the official European Union method and following amendments [5,6], the legal quality characteristics, such as peroxide value (meq O_2_ kg^−1^); acidity (% oleic acid); and UV spectroscopic indexes (K232, K270, and ∆K), were measured [29].

Volatile compound contents were measured as described in Fortini, Migliorini, Cherubini, Cecchi, and Calamai (2017) [30], using the HS-SPME-GC-MS technique. Compounds were identified and quantified (mg/kg) by comparison of their mass spectra and retention times with those of the internal standard mixture (ISTD MIX), consisting of the following 11 compounds: 4-methyl-2-pentanol, 3,4-dimethylphenol, 1-butanol-d10, hexanoic acid-d11, ethyl hexanoate-d11, ethyl acetate-d8, toluene-d8, 6-chloro-2-hexanone, acetic acid-2,2,2-d3, 3-octanone, and trimethylacetaldehyde.

Briefly, the analyses were carried out by weighing, into 20-mL screw cap vials fitted with a PTFE/silicone septa, 4.3 g of an oil sample and 0.1 g of an internal standard mixture. After 5 min equilibrium at 60 °C, a SPME fibre (50/30-µm DVB/CAR/PDMS by Supelco, St. Louis, UK) was exposed for 20 min in the vial headspace under orbital shaking (500 rpm). Then, the fibre was immediately desorbed for 2 min in a gas chromatograph injection port operating in splitless mode at 260 °C. The identification of volatile compounds was performed by gas chromatography coupled to quadrupole-mass spectrometry using an Agilent GC-MS 7890B-5977E, equipped with an Innowax capillary column (50 m × 0.4 id × 0.4 um ds). Initial column temperature was held at 40 °C for 10 min, then increased to 200 °C at 5 °C/min, then to 260 °C at 10 °C/min, and finally, to 250 °C at 10 °C/min, with a hold time of 4 min. Helium was used as the carrier gas at 1.2-mL/min constant flow. The temperature of the source was 230 °C, while the transfer line was 250 °C. The mass detector was operated in scan mode within a 29-330-Th mass range at 1500 Th/s, with an IE energy of 70 eV. Volatile compounds quantified with this method were: heptane, octane, methyl acetate, 2-butanone, ethyl acetate, methyl propanoate, 2-methyl batanal, isovaleraldehyde, ethyl propanoate, 3-pentanone, valeraldehyde, ethyl vinyl ketone, 2-butanol, ethyl butanoate, propanol, butyl acetate, hexanal, isobutanol, 2-pentanol, 2-pentenal, Z-3-hexenal, 1-penten-3-ol, 1-penten-3-one, 2-heptanone, heptanal, limonene, 2-methyl-1-butanol + 3-methyl-1-butanol, 2-methyl-1-butanal + 3-methyl-1-butanal E-2-hexenal, ocimene, pentanol, hexyl acetate, 2-octanone, octanal, 1-octen-3-one, E-2-penten-1-ol, Z-3-hexenyl-acetate, E-2-heptenal, 2-heptanol, Z-2-pentenol, E-2-hexenyl-acetate, 5-hepten-2-one-6-methyl, 1-hexanol, E-3-hexen-1-ol, Z-3-hexen-1-ol, 2-nonanone, nonanal, 2,4-hexadienal, E-2-hexenol, Z-2-hexenol, 2-octanol, E-2-octenal, 1-octen-3-ol, heptanol, 2,4-heptadienal, decanal, benzaldehyde, E-2-nonenal, propanoic acid, octanol, butanoic acid, E-2-decenal, nonanol, 2,4-nonadienal, pentanoic acid, 2,4-decadienal, hexanoic acid, guaiacol, phenyl ethanol, phenol, 4-ethylguaiacol, 4-ethyl-phenol, phenol-4-ethyl-2-methoxy, and phenol-2-mehoxy.

### 2.4. Turbidity Grade

A Hach 2100 turbidimeter (Hach, Loveland, CO, USA) was used to measure the olive oil sample turbidity grades in nephelometric turbidity units (NTU).

About 25 g of the oil sample was taken from the bottle, previously shaken for 1 min, and put in a standard glass vessel well-cleaned, which was then inserted in the closed vessel chamber of the turbidimeter. Turbidity grade was measured at equilibrium after approximately one minute.

### 2.5. Water Content and Water Activity

Water content (%*w/w*) was analysed with a HYDRANAL™-Moisture Test Kit (Honeywell Fluka™ 37858, Bucharest, Romania), which is a Karl Fischer Kit for visual water determination without titration. Exactly 1 mL of olive oil sample was dissolved in neutralised HYDRANAL™-Solvent E, and the titrating reagent (HYDRANAL™-Titrant 5E) was added until the equivalence point was reached. Water content was quantified as % water content weight/ 100 g olive oil sample (%*w/w*)

A Rotronic Hygroskop DT hygrometer (Michell Italia Srl, Milan, Italy) was used to measure the water activity value (Aw). Approximately 6.5 mL of olive oil samples were placed in standard sample cups. At equilibrium, after approximately 30 min, the water activity value was measured.

### 2.6. Solid Particles Content

Solid particles content was measured using the method described in Zullo and Ciafardini (2018) [31]. Specifically, 5 g of olive oil previously filtered through a 0.45-µm nitrocellulose membrane, so lacking in solids content, was vacuum-filtered to saturate a Whatman grade 1 filter paper (Merck, Darmstadt, Germany). The same filter paper, soaked with oil, was weighed with an analytical balance to determine the tare and used to filter approximately 10–15 g of the oil sample. After the oil sample vacuum filtration, the filter was weighed again. Solid particles content was calculated as the difference in weight and quantified as % solid particles weight/ 100 g olive oil sample (%*w/w)*.

### 2.7. Microbial Analyses

Microorganism enumeration was performed according to the method reported in Zullo, Cioccia and Ciafardini (2010) [32] with some modifications: an aliquot of a sample (approx. 20 mL) was taken from each bottle, previously shaken for 1 min, under sterile conditions and filtered through a sterile 0.45 µm nitrocellulose membrane. Then, the membrane was transferred into a 50 mL sterile Falcon tube containing 20 mL of sterile physiological solution (NaCl 0.85%) and homogenised with an Ultra Turrax homogeniser (T25, IKA, Milan, Italy) for 1 min. Next, 200 μL aliquots of serial dilutions of each homogenised sample were plated onto YPD agar medium (Carlo Erba Reagents, Milan, Italy).

After 48–72 h incubation at 28 °C, colonies with different morphologies were counted and recorded. According to our experience, the limit of quantification for the microbial analyses is 0.2 log CFU/g.

### 2.8. Sensory Analyses

A simplified version of the International Olive Council (IOC) sensory panel test was applied [33]. Trained judges were asked only to smell olive oil samples and give a score on a scale of 1–9, as described by the official IOC method. First, they assessed the fruity attribute. Then, they were asked to indicate if one of the sensory defects described in the official IOC method was present. If so, they were asked to state which one and score. Sensory analyses were carried out for each storage time, beginning at time 0.25 (i.e., 6 h after the extraction). The panel was made up of three women and five men, all trained following the official IOC procedure [34]. The panellists, aged from 29 to 58 and all non-smokers, worked all for the Taste Commission of The Ministero delle Politiche Agricole, Alimentari, Forestali e del Turismo (MIPAAAFT—Italian Ministry of Agri-Food and Forestry Policy and Tourism).

### 2.9. Statistical Analyses

A linear model that included the two tested variables (filtration and storage time) and their interactions was used to fit the experimental data. Filtration was treated as a categorical variable (Yes or No), while time was considered as a continuous variable, modelled between 0 and 30 days of storage. Data were analysed with R software (The R Foundation for Statistical Computing, Vienna, Austria). A two-way ANOVA was performed in order to assess the significant differences (*p* < 0.05). Following Dunn and Smyth (2018) [35], nonsignificant terms were removed, and then, the model was checked again.

## 3. Results and Discussion

### 3.1. Turbidity Characterisation, Microbial Contamination, and Legal Requirements

Filtration caused a deep change in the treated olive oil samples. The water content fell from 0.40 ± 0.05 %*w/w* to 0.07 ± 0.02 %*w/w*, while the solids decreased from 0.28 ± 0.08 %*w/w* to 0.03 ± 0.03 %*w/w*. These results are consistent with all of the studies in the literature that have applied depth filtration [20,21,27]. The turbidity fell from 2642 ± 174 NTU to 18 ± 4 NTU, and the water activity (aw) decreased from 0.70 ± 0.03 to 0.42 ± 0.04. These results are also consistent with the literature [11,22,26] regarding aw values and the turbidity grade of filtered oil.

In the water-in-oil emulsions, like veiled olive oils, microorganisms (like yeasts and moulds) are dispersed within the microdroplets of water, because the oil matrix does not allow their survival and growth. For this reason, the removal of water microdroplets by filtration led to a statistically significant (*p*-value < 0.001) decrease in the microbial content. Specifically, the microorganisms grown on YPD agar medium fell from 3.8 ± 0.2 log CFU/g in veiled oil samples to undetectable in filtered samples (Table 1). These results agree with those reported in the literature [20,22,31,32]. This difference was still observed after 30 days of storage. Microbial survival in veiled oil samples can be explained by the following factors: (i) the dispersion of microorganisms in water, which is a good environment for microbial survival, (ii) the presence of insoluble solids, which are rich in microorganism nutrients, and (iii) a water activity value > 0.6, which supports microbial survival and enzyme activity [36,37].

All samples were characterised by chemical indexes that were well within the legal limits for the definition of extra virgin olive oil (Table 1). During storage, no differences emerged in the veiled and filtered olive oil samples. The filtered and veiled oil samples had similar values for acidity, K232, and ∆K. Filtration takes to a small and not statistically significant (*p*-value > 0.05) increase in the peroxide number and to a decrease in the K270 value. Therefore, although auto-oxidation phenomena were slightly affected by the filtration treatment, they were negligible over time in the tested storage conditions.

### 3.2. Sensory Attributes

**Figure 1:**
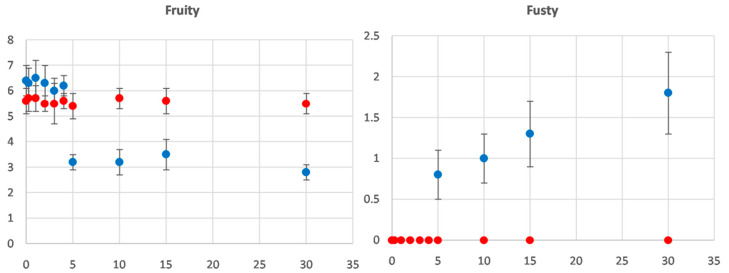
Mean contents of the “fruity” attribute and “fusty” defect scores in veiled (blue circles) and filtered (red circles) olive oil samples during storage.

In Figure 1, the evolution of the “fruity” attribute and “fusty” defect during 30 days of storage is reported. Up to four days of storage, judges were unable to detect any sensory defects in both filtered and veiled olive oil samples. However, filtered samples were perceived as less fruity than veiled samples (an average of 0.8 fewer points on a 9-point scale), which is consistent with the literature [26,38,39]. After five days of storage, our judges started to perceive the fusty defect in veiled samples (0.8 ± 0.3 intensity score) and not in filtered samples. At the same time, they started to describe filtered samples as fruitier than veiled samples. In Figure 1, it is possible to observe that the “fruity” score of veiled olive oil samples statistically significantly decreases (*p*-value < 0.05); instead, the “fruity” score of filtered olive oil samples do not change during 30 days of storage time. We can hypothesise that the appearance of the fusty defect caused the decrease in the fruity score of the veiled oils. Since olive oil is considered to be of the “extra virgin” category when the median of the defects is equal to zero [33], in our experiment, the veiled samples were downgraded from “extra virgin” to “virgin” olive oil after five days of storage. Between five days and 30 days of storage, judges noted a further increase in the fusty defect, but the 3.5-limit value for downgrading the sample to the “lampante” olive oil category was not reached. No rancid defect was perceived.

### 3.3. Volatile Compound Contents

We assessed the effects of filtration, storage time, and their interactions. A significant effect of storage time was found to be consistent with a significant change in the volatile compound contents, independent of the filtration treatment. On the other hand, a significant effect of filtration was found to be consistent with a significant difference in the volatile compound contents, and this difference remained stable during storage. Finally, we found a significant interaction between filtration and storage time. Here, specific compounds changed over time, and the change was linked to the filtration treatment. The interaction allowed us to evaluate the very important evolution of different volatile compounds in veiled and limpid samples.

#### 3.3.1. Pleasant LOX Pathway Volatile Compound Contents

Experimental data has identified several statistically significant differences (*p*-value < 0.05) in the LOX pathway [7,8,12,40,41,42,43] related to the filtration treatment, storage time, and their interactions.

**Figure 2:**
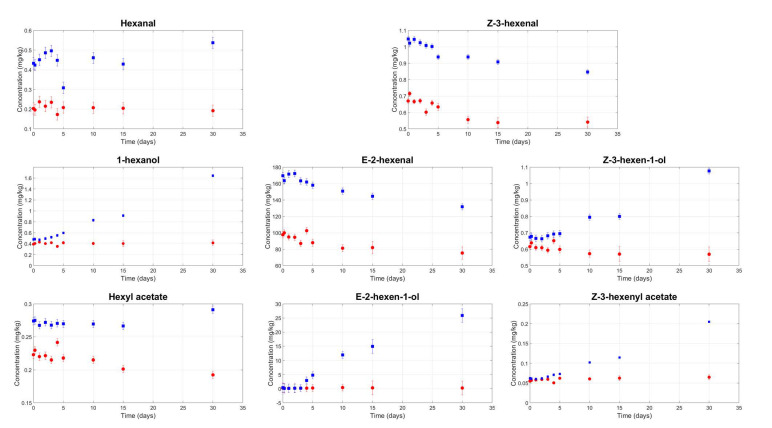
Mean contents of the lipoxygenase (LOX) compounds for the C6 branch in veiled (blue squares) and filtered (red circles) olive oil samples during storage.

**Figure 3:**
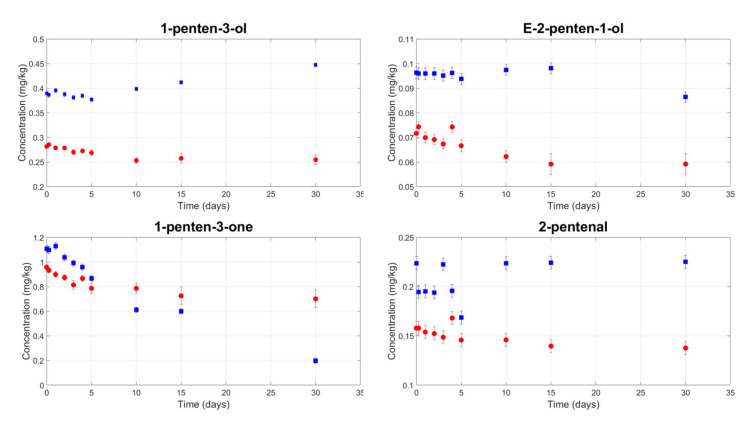
Mean contents of the LOX compounds for the C5 branch in veiled (blue squares) and filtered (red circles) olive oil samples during storage.

Figure 2 and Figure 3 show the kinetics of LOX volatile compounds from the C6 and C5 branches, respectively. In all samples, there is a statistically significant decrease in the LOX volatile compound contents as a result of filtration; the ANOVA highlighted a significant (*p*-value < 0.05) main effect of filtration. On average, 27.3% of the LOX compounds were removed by filtration, but this varied as a function of the chemical properties of specific compounds. The smallest decrease was found for Z3-hexen-1ol (−8.5%), compared to −53.1% for hexanal. These observations are consistent with data reported in the literature [44] and could explain the less fruity perception of the filtered samples compared to the veiled samples measured by the panel test at the beginning of storage.

A statistically significant interaction between filtration and storage time (*p*-value < 0.05) was found for the following LOX volatile compounds: 1-hexanol, E2-hexen-1-ol, Z3-hexen-1-ol, Z3-hexenyl acetate, and 1-penten-3-ol. All showed the same behaviour. After a specific storage time, the organic compound content in the veiled oil samples started to increase, while it remained constant in the filtered samples. For example, the 1-hexanol content remained at 0.4–0.5 mg/kg until the fifth day of storage in both the filtered and veiled samples. However, at 30 days, it reached 1.7 mg/kg in veiled oils compared to 0.4 mg/kg in filtered oils. Similarly, the E2-hexen-1-ol content was below the detection threshold until the third day of storage in all samples but reached a mean of 25 mg/kg at the end of storage for the veiled oils. This behaviour could be related to the enzymes that are responsible for the LOX pathway. They remain active in veiled samples thanks to the residual water content and high water activity; however, they are inhibited in filtered samples due to the almost complete absence of water and low water activity.

Some authors have claimed that the increase in LOX compounds in veiled oil samples is responsible for an increase in the pleasant fruity attribute; on the other hand, other research has found that several of the compounds that increased in veiled oils, which are considered as positive at low concentrations, have an unpleasant odour at higher concentrations. Specifically, 1-hexanol has been perceived as “rough mouthfeel and rancid”; E-2-hexen-1-ol as “wine-like, undesirable”; Z-3-hexen-1-ol, Z-3-hexenyl acetate, and 1-penten-3-ol as “wet earth, undesirable”; and 1-penten-3-one as “unpleasant” [12,40,41,43].

We were unable to establish whether the observed changes in the volatile compound contents represented an improvement or a deterioration in the odour of veiled oil, as our panel test did not perceive any of the above defects. Nor did it reveal any significant increase in the fruity attribute as the storage time increased. It is possible that the formation of the fusty defect (see next paragraph) masked the increase in the fruity attribute to the point that the filtered samples appeared fruitier than the veiled samples.

#### 3.3.2. Unpleasant Volatile Compound Contents

**Figure 4:**
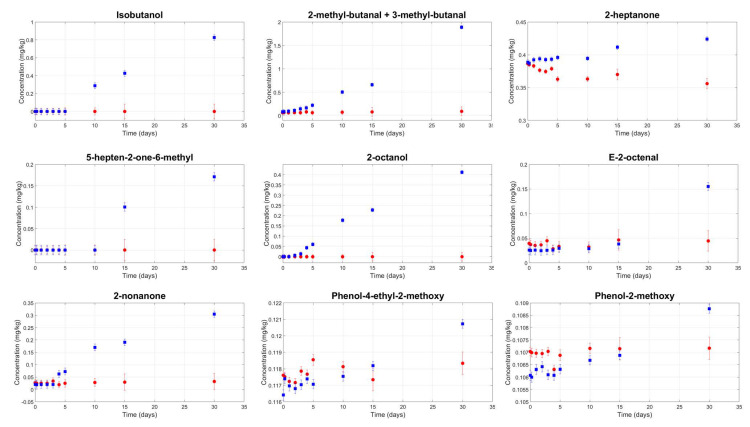
Mean contents of unpleasant volatile organic compounds in veiled (blue squares) and filtered (red circles) olive oil samples during storage.

Unpleasant volatile compounds were found in all samples, and their kinetics were measured during storage time (Figure 4).

A statistically significant interaction between filtration and storage time (*p*-value < 0.05) was found for the isobutanol, 2-methyl-butanal, and 3-methyl-butanal compounds. These compounds are related to microbial amino acid metabolism and are derived from valine, isoleucine, and leucine, respectively [45]. Their contents increased during storage in the veiled samples. In the filtered samples, the initial content was low and did not increase during storage. The same significant interaction between filtration and storage time was observed for two volatile phenol compounds—namely, phenol-2-methoxy and phenol-4-ethyl-2-methoxy. According to the literature, the former is related to the metabolic activity of several yeasts [46], while the latter is usually related to Brettanomyces contamination [47]. Since cinnamic acid is the precursor of phenol-4-ethyl-2-methoxy, which is an olive oil biophenol [48], the high experimental microbial content in the veiled oil samples could explain both its presence and its increase with storage time.

Similarly, a significant interaction between filtration and storage time was observed for the following five volatile compounds: 2-heptanone, 5-hepten-2-one-6-methyl, E-2-octenal, 2-octanol, and 2-nonanone. In this case, the contents were initially the same in both the filtered and veiled samples, but after a few days, the contents increased in the veiled samples. The detection threshold was exceeded after a storage time ranging from three days (for 2-octanol) to 15 days (for E-2-octenal and 5-hepten-2-one-6-methyl).

According to data reported in the literature [39,49] the different behaviours of the 10 volatile compounds in the veiled and filtered samples could be caused by both microbial contamination and factors promoting microbial activity, such as oil turbidity, water and solid contents, and water activity. The high turbidity and high water activity (in the veiled samples) that promoted volatile compound formations were consistent with the high microbial contents; on the other hand, low turbidity and low water activity (in the filtered samples) were consistent with an undetectable experimental microbial count, and the volatile compounds remained constant during storage.

All 10 volatile compounds are unpleasant in an olive oil and are frequently related to the fusty and other sensory defects. For example, 2-heptanone, 2-nonanone, and 5-hepten-2-one-6-methyl have been related to the “mustiness-humidity” defect and 2-octanol to the “earthy” and “mustiness-humidity” defects [2,8]. The fusty attribute perceived by the panel test after five days in veiled samples is consistent with the experimental volatile compound kinetics (Figure 4).

**Figure 5:**
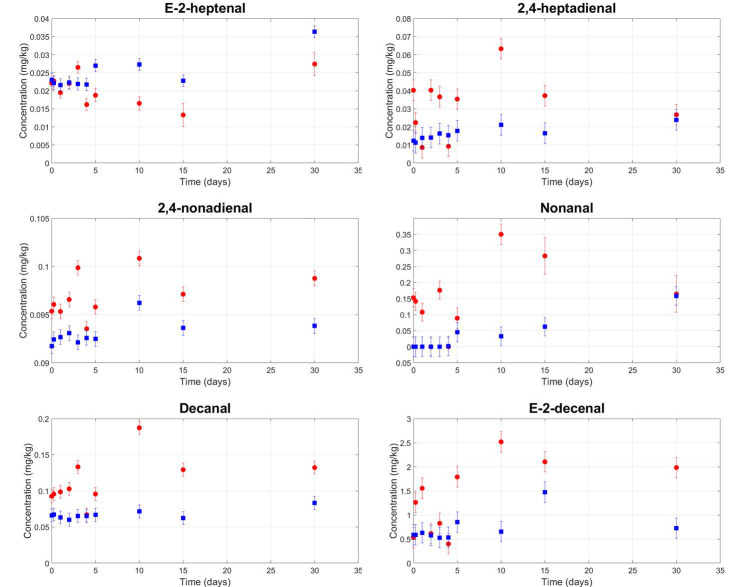
Mean contents of the volatile compounds related to the rancid defect in veiled (blue squares) and filtered (red circles) olive oil samples during storage.

Other volatile compounds, typically related to the rancid defect, have also been observed [50,51,52]. A significant main effect of filtration has been found for 2,4-heptadienal, E-2-hepteneal, 2,4-nonadienal, nonanal, decanal, and E-2-decenal. In our experiment, their contents slightly increased as an immediate effect of filtration (Figure 5), but we found no significant interaction between the filtration and storage time. It seems that, as reported in the literature [19,27,53,54,55] filtration resulted in little oxidation of the oil samples in our experiment. The panel test supports the hypothesis of limited auto-oxidation, as no rancid defect was perceived. However, Guerrini et al. (2020) [11] argue that one month of storage is insufficient to be able to observe the development of the rancid defect.

## 4. Conclusions

The focus of this original study was to optimise the scheduling of filtration during the olive milling season. Consequently, during the first month of storage, we examined the kinetics of volatile compound contents in immediately filtered olive oil samples and in the respective veiled olive oil samples, which would be hypothetically filtered after several days.

Two effects were observed on the olive oil quality. First, the veiled oil samples were downgraded from the “extra virgin” to “virgin” quality category after less than five days in protective storage conditions. This deterioration was caused by the formation of unpleasant volatile compounds and an increasing perception of the “fusty” defect during storage, probably due to undesirable oil-born microorganisms. Second, lipoxygenase volatile compound contents were highest in the veiled olive oil samples, and the positive fruity sensory attribute was most marked in the veiled olive oil samples at the beginning of storage. It is possible that the appearance of the “fusty” defect could have masked an increase in the fruity attribute observed at the beginning of storage. Our experiment showed that a fast filter press filtration prevented microbial contamination and limited microbial and enzymatic activity. Consequently, the filtered olive oils samples were not downgraded, as the “fusty” defect did not develop; this critical, positive result outweighs the disadvantage of a less fruity olive oil.

The obtained results allowed to increase the general knowledge of the volatile compositions of filtered and unfiltered olive oils during the first month of storage and to confirm the stabilisation role of filtration due the removal of water, solids, and microorganisms, limiting the microbial and enzymatic activity. Indeed, the microbial and endogenous enzyme activity, responsible for the development of volatile compounds related to defects like “fusty” and “muddy”, were promoted by factors such as the oil turbidity, water and solid contents, and high water activity.

The comparison between the kinetics of the volatile compound contents in the veiled and filtered olive oil samples has shown not only that unfiltered oils deteriorate more than filtered ones but, also, that this deterioration is really fast. The innovative take by this work is an operative contribution to optimise the workload during olive oil production. Our study demonstrates that filtration should be carried out within a few days after olive oil production to reduce the risk of the emergence of sensory defects. Furthermore, a trade-off between workload and quality risk was highlighted. Immediately after the production, the workload is at its peak, while the risk for quality is minimum. Then, the workload progressively decreases, while the risk increases until the commercial category downgrade. According to our data, the filtration had to be done in five days from the production. This value could change according to the turbidity composition of the olive oil, but only in the first few days did we find a low risk for extra virgin olive oil quality.

## Figures and Tables

**Table 1:** Legal limits for the chemical characteristics and mean microbial counts for olive oil samples after milling or filtration (t_0_) and after one month of storage (t_1_). F refers to filtered oil samples, and V refers to veiled oil samples.

	*F*		*V*		
	*t_0_*	*t_1_*	*t_0_*	*t_1_*	*Legal Limits for “Extra Virgin” Category* [5,6]
Acidity (% oleic acid)	0.35 ± 0.09 ^ax^	0.36 ± 0.10 ^ax^	0.32 ± 0.02 ^ax^	0.33 ± 0.03 ^ax^	≤0.8
Peroxide value (meqO_2_kg^−1^)	6.2 ± 0.7 ^bx^	6.2 ± 0.8 ^bx^	5.8 ± 0.3 ^ax^	5.9 ± 0.4 ^ax^	≤20
K232	1.59 ± 0.06 ^ax^	1.60 ± 0.05 ^ax^	1.59 ± 0.07 ^ax^	1.62 ± 0.07 ^ax^	≤2.50
K270	0.09 ± 0.01 ^bx^	0.09 ± 0.02 ^bx^	0.18 ± 0.01 ^ax^	0.18 ± 0.01 ^ax^	≤0.22
∆K	−0.004 ± 0.000 ^ay^	−0.001 ± 0.002 ^ax^	−0.004 ± 0.001 ^ax^	−0.003 ± 0.003 ^ax^	≤0.01
Microbial cell count (log CFU/g)	0.6 ± 1.0 ^bx^	0.0 ± 0.0 ^bx^	3.8 ± 0.2 ^ax^	3.5 ± 0.3 ^ax^	-

a and b indicate significant differences (*p* < 0.05) as a function of the treatment (with or without filtration), while x and y indicate significant differences (*p* < 0.05) as a function of the storage time. The legal limits of microbial cell count for “extra virgin” olive oil category is not reported in literature.
